# Unsupervised learning of facial emotion decoding skills

**DOI:** 10.3389/fnhum.2014.00077

**Published:** 2014-02-27

**Authors:** Jan O. Huelle, Benjamin Sack, Katja Broer, Irina Komlewa, Silke Anders

**Affiliations:** ^1^Social and Affective Neuroscience, Department of Neurology, Universität zu LübeckLübeck, Germany; ^2^School of Ophthalmology, South West Peninsula Postgraduate Medical EducationPlymouth, UK

**Keywords:** dynamic facial expressions, emotional facial expressions, unsupervised learning, perceptual learning, social learning, cross-cultural learning, empathy

## Abstract

Research on the mechanisms underlying human facial emotion recognition has long focussed on genetically determined neural algorithms and often neglected the question of how these algorithms might be tuned by social learning. Here we show that facial emotion decoding skills can be significantly and sustainably improved by practice without an external teaching signal. Participants saw video clips of dynamic facial expressions of five different women and were asked to decide which of four possible emotions (anger, disgust, fear, and sadness) was shown in each clip. Although no external information about the correctness of the participant’s response or the sender’s true affective state was provided, participants showed a significant increase of facial emotion recognition accuracy both within and across two training sessions two days to several weeks apart. We discuss several similarities and differences between the unsupervised improvement of facial decoding skills observed in the current study, unsupervised perceptual learning of simple visual stimuli described in previous studies and practice effects often observed in cognitive tasks.

## INTRODUCTION

Dating from Darwin’s notion that “the different races of man express their emotions […] with remarkable uniformity” (Darwin, 1872) facial expressions of emotion have long been viewed as a hard-wired product of evolution that is universally understood across human cultures and, to some extent, even mammalian species. Although most researchers now agree that human emotional facial expressions can vary considerably across social groups and cultures (for a meta-analysis see Elfenbein and Ambady, 2002), few studies have aimed to systematically investigate how encoding and decoding of facial expressions is shaped by social learning. Furthermore, the majority of studies that did investigate learning of facial emotion recognition aimed to develop training programs that might improve the participants’ social or inter-cultural skills and therefore mixed different types of training (e.g., McAlpine et al., 1992; Stewart and Singh, 1995; Bölte et al., 2002; Silver et al., 2004; Solomon et al., 2004; Wölwer et al., 2005; Matsumoto and Hwang, 2011).

Theoretical work has suggested that associative learning during infancy might play an important role in the acquisition of facial decoding skills. The reasoning is that because infants are often exposed to similar emotional contexts as their mothers, the sight of their mother’s facial expression in a given context becomes gradually associated with the infant’s own emotional state in that context through Hebbian learning. Such associative learning, it is argued, can take place even if the infant’s and the mother’s emotional state are different because the mothers often mirror the infant’s emotional state (Keysers and Perrett, 2004; Keysers and Gazzola, 2006). It has further been proposed that once these links have been established, contextual cues might be sufficient to fine-tune associations between observed facial expressions and emotional meaning. Indeed, the few studies that have systematically investigated learning of facial emotional recognition provide evidence that facial decoding skills can be sharpened both in adults (Elfenbein, 2006) and children (Beck and Feldman, 1989) if appropriate information about the affective content is provided on a trial-by-trial basis.

While such information might often be available during normal infant development, it will often be absent in adult life. Consider, for example, an individual observing the expressive emotional behavior of members of a different social group or culture. For this individual the emotion giving rise to the emotional display might be as obscure as the behavior itself. Thus, if cross peer-group and cross-cultural learning of facial emotional expressions can take place across the life span as suggested by the works by Elfenbein and others (Elfenbein and Ambady, 2002; Elfenbein, 2006), then some form of learning that does not rely on an external teaching signal might be effective in this learning.

The neural processes and mechanisms underlying unsupervised improvement of stimulus perception have extensively been studied in vision research. These studies provide consistent evidence that repeated exposure to simple visual stimuli such as tilted lines can lead to enhanced stimulus detection, discrimination or categorization in the complete absence of an external teaching signal (e.g., Poggio et al., 1992; Crist et al., 1997). A well-known example for this is the texture discrimination task in which participants learn to judge the orientation of a simple target stimulus (a number of aligned lines) among a number of distracter lines (Karni and Sagi, 1991). Interestingly, two recent studies that aimed to show that training with appropriate feedback can improve emotion recognition skills provided evidence that emotion recognition learning does not only take place if participants receive appropriate feedback, but might also occur in the complete absence of feedback (Blanch-Hartigan, 2012; Hurley, 2012).

Here, we provide further evidence that mere practice without an external teaching signal can improve facial emotion decoding skills in adults. In addition, we explore whether interpersonal traits can explain interindividual differences in learning. During two training sessions several days to weeks apart, participants saw video clips of dynamic facial expressions of five different women and were asked to decide which of four possible emotions (anger, disgust, fear, and sadness) was shown in each video. Although no information about the correctness of the participant’s response or the woman’s true affective state was provided, participants showed a significant increase of facial emotion recognition accuracy both within and between training sessions. This effect was modulated by stimulus duration and interpersonal traits. We discuss several similarities and differences between the unsupervised learning of facial decoding skills observed in the current study, unsupervised perceptual learning of simple visual and auditory stimuli described in previous studies and practice effects often observed in cognitive tasks.

## MATERIALS AND METHODS

### ETHICS STATEMENT

Participants gave their informed consent before participation according to the guidelines of the American Psychological Association (http://www.apa.org/ethics) and the study was approved by the Ethics Committee of the Universität zu Lübeck. All data were analyzed anonymously.

### PARTICIPANTS

Forty female participants were recruited from the Universität zu Lübeck, Germany. All participants were German-speaking Caucasians and none of the participants reported current or previous neurological or psychiatric illnesses. To investigate possible effects of the duration of the consolidation interval between the first and the second training session on learning, half of the participants had their second training sessions 2 days after the first training session (*2-days consolidation interval*), the other half 40–80 days (mean 59 days) after the first training session (*2-months consolidation interval*). Two participants were not available for the second training session; data of these participants were excluded from the analysis. The final sample consisted of 38 participants (20 with a 2-days consolidation interval, 18 with the 2-months consolidation interval) with an average age of 22.2 years (range 19–28 years).

### ASSESSMENT OF INTERPERSONAL TRAITS

To examine possible relations between interpersonal traits and improvement of facial decoding skills participants completed the German 16-item version of the *Interpersonal Reactivity Index* (IRI, Davis et al., 2003), the *Saarbrücker Persönlichkeitsfragebogen* (SPF, http://psydok.sulb.uni-saarland.de/volltexte/2009/2363/) after the first training session. The IRI assesses the participant’s interpersonal traits on four different subscales: spontaneous attempts to adopt the perspectives of other people (perspective-taking), tendency to identify with characters in movies, novels, plays, and other fictional situations (fantasy scale), feelings of warmth, compassion, and concern for others (empathic concern) and feelings of anxiety and discomfort when observing another’s negative experience (personal distress).

### STIMULI

In order to investigate subtle changes of ecologically valid facial emotion decoding skills we sought to use a stimulus set in which (i) senders expressed their true emotional state (rather than just showing a given prototypical facial expression) and (ii) senders communicated their true emotional state to a socially significant person (rather than just looking into a camera). Thus, we used video clips recorded in a previous fMRI (functional magnetic resonance imaging) study in which participants (senders) were asked to imagine and submerge themselves into a cued emotional situation and to facially express their feeling to their romantic partner who they believed was observing them online via a video camera (Anders et al., 2011). Analysis of the data from that study showed that observers were not only able to identify the sender’s emotional state above chance at the behavioral level, but that showing and observing a given emotion evoked emotion-specific patterns of brain activity that were highly similar in the sender’s and the observer’s brain (Anders et al., 2011). For the current study, we selected videos clips of anger, disgust, fear, and sadness, each expressed by five different female Caucasian senders. These clips were selected from eight videos (two per emotion) recorded from each sender, whereby each video comprised four 20 s periods of a given emotion, separated by 20 s neutral periods. Only negative emotions were selected to avoid ceiling effects introduced by joy (which is usually very easily recognized among the negative emotions).

In order to permit the investigation of possible effects of stimulus duration on learning, videos were cut into clips of five different lengths (2 s, 4 s, 6 s, 8 s, and 10 s), each beginning with the onset of an emotional period. The final set of 100 different video clips contained one sample of each sender-by-emotion-by-length combination. These video clips were shuffled and divided into five subsets of twenty video clips, with the restriction that each subset contained one sample of each length-by-emotion combination and one sample of each sender-by-emotion combination. Subsets were presented in a counterbalanced order across participants, and a different order was used for the first and second training session of each participant. Analysis of hit rates for the five subsets during the first training session revealed no significant difference between stimulus subsets (one-way ANOVA with factor stimulus set, *F*[4,148] = 1.4, *p* = 0.23), indicating that facial expressions were evenly distributed across stimulus sets with regard to emotion recognition difficulty.

### PROCEDURE

**FIGURE 1:**
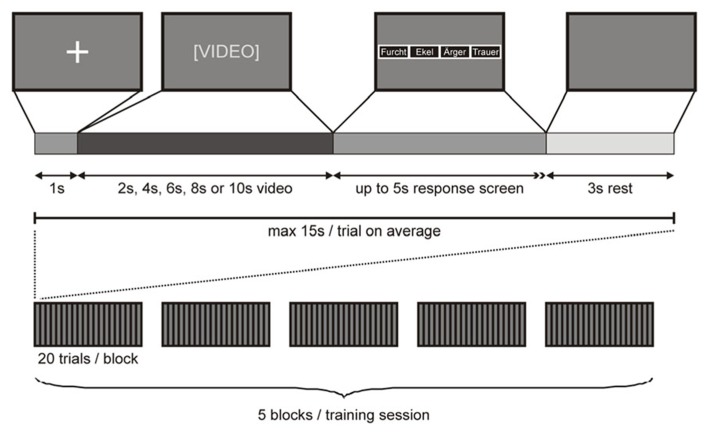
**Stimulus presentation.** Top row: A fixation cross on dark background signaled the beginning of a trial. After 1 s, the fixation cross screen was replaced with a video of 2 s, 4 s, 6 s, 8 s, or 10 s length. Immediately after the video, a response screen appeared with four small boxes, each labeled with one emotion (whereby the order of the labeled boxes on the screen corresponded to the order of the response buttons on the keyboard) indicating the participant to convey her decision by button press (maximal response interval of 5 s). As soon as the participant had conveyed her response, the response screen was replaced with a dark screen for 3 s, after which the next trial began. Bottom row: A complete training session comprised five blocks of 20 trials. Each block of 20 trials contained a different subset of video clips.

Participants were tested in two training sessions, either 2 days or 40–80 days (mean 59 days) apart (see above). Video presentation during each training session was divided into five blocks, each containing one subset of video clips. Video clips were presented on a 15″2 TFT laptop screen approximately 500 mm in front of the participant’s face. Each video clip was preceded by a 1 s fixation cross on a dark background. Immediately after the video clip, a response screen appeared with four small boxes, each labeled with one emotion ( “anger”, “disgust”, “fear”, “sadness”), indicating the participant to convey her decision by button press. Four keys on the keyboard (*D*,* G*,* J*,* L*), each labeled with one emotion, were used as response buttons (whereby the order of the labeled boxes on the screen corresponded to the order of the response buttons on the keyboard). As soon as the participant had entered her response (maximal response interval of 5 s), the response screen was replaced with a dark screen for a fixed intertrial interval of 3 s. Importantly, the assignment of response buttons was counterbalanced across participants and a different assignment was used for the first and second training session for each participant. A response was defined as correct if the response button pressed by the participant corresponded to the emotion cued to the sender and as incorrect otherwise. A missing response was counted as an incorrect response. The presentation of a complete subset of video clips took a maximum of 20 × 15 s = 5 min, depending on the participant’s response time. After each of these blocks, a short break was inserted (< 3 min), resulting in a maximum duration of 5 × 8 min = 40 min for each training session (**Figure 1**).

To familiarize participants with the experimental setting, each training session was preceded by three practice trials with video clips of a sender that was not used in the main experiment. Stimulus presentation and response logging were implemented with Presentation software (Neurobehavioral Systems Inc., Albany, CA, USA).

### DATA ANALYSIS

First, emotion recognition data were reduced by computing average hit rates and response times for each block and participant. Second, to obtain an estimate of initial performance and block-to-block increase (hit rates) or decrease (response times) of performance during each training session for each participant, a straight line with slope *b*_j_ and constant *c*_j_ was fitted through block averages, separately for each training session, using the least square criterion such that

$$y_{ji}=b_{j}.x_{ji}+c_{j}+e_{ji},\,withi=1,2,...,5\,andj=1,2$$

where *y*_ji_ is the estimated hit rate in block *i* of training session *j*, *x*_ji_ is the mean-corrected number of block *i* of training session *j*, and *e*_ji_ is the error in block *i* of training session *j*.

In our main analysis, we then tested (i) whether learning slopes (*b*_1_,*b*_2_) were larger (hit rates) or smaller (response times) than zero (indicating learning within training sessions) and (ii) whether there was a significant increase (hit rates) or decrease (response times) of estimated performance from the first block of the first training session to the first block of the second training session (*y*_2,1_ - *y*_1,1_) (indicating consolidation across training sessions). To test for consolidation across training sessions, we used estimated hit rates/response times during the first block of each training session (*y*_1,1_ and* y*_2,1_) rather than average performance during each session because they represent unbiased estimates of performance at the *beginning* of each training session.

For hit rates, we performed three additional analyses. First, to examine whether stimulus duration had an effect on learning, we tested for differences in initial performance (*y*_1,1_), learning slopes (*b*_1_, *b*_2_), and consolidation (*y*_2,__1_ - *y*_1,1_) between short and long video clips. For this analysis, the parameters *b* and *y* were computed as described above, but this time separately for short videos (2–4 s) and long videos (8–10 s).

Second, to test for possible relations between interpersonal traits and (learning of) facial decoding skills, we correlated each participant’s initial performance (*y*_1,1_) and average learning slopes (*b*_1_ + *b*_2_) with her scores on the four IRI subscales (fantasy, empathic concern, perspective taking, personal distress).

Finally, we asked whether learning differed across emotions. Because of the limited number of trials per emotion, data were averaged across the five blocks of each training session for this analysis. Because hit rates for single categories can be affected by response biases, we computed average unbiased hit rates *hu*_j_, _e_ (Wagner, 1993), *hu*_j_, _e_ = (# of hits × # of hits)/(# of responses × # of stimuli) for each emotion and training session, where *hu*_j_, _e_ is the unbiased hit rate for emotion *e* in training session *j*. Differences between emotions were assessed by a four-by-two ANOVA with factors emotion and training session.

Student’s *t*-test was used to test for differences unless otherwise indicated. In cases where we had a one-sided hypothesis, statistical tests were performed one-tailed, in all other cases two-tailed.

## RESULTS

### MAIN ANALYSIS

**FIGURE 2:**
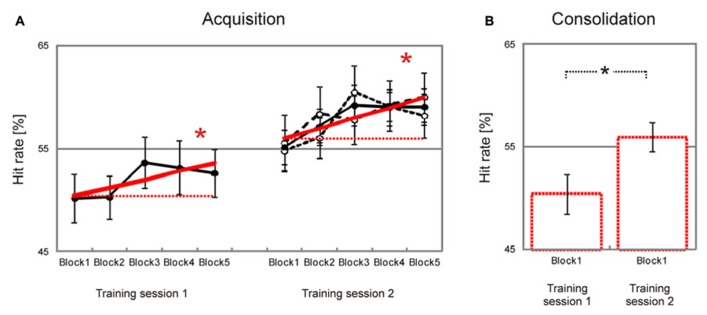
**Improvement of facial emotion recognition accuracy.** Participants showed a significant linear increase in emotion recognition accuracy within both training sessions (bold red lines in **(A)**. Emotion recognition accuracy also increased significantly from the first block of the first training session to the first block of the second training session, indicating consolidation of emotion recognition accuracy across training sessions (dashed red lines in **(A)**, bar charts in **(B)**. Filled circles in **(A)** represent block averages across all participants. Learning and consolidation did not differ between participants who had a two-day interval between the two training sessions (open circles and narrow dashed black line in **(A)**, only shown for the second training session) and participants who had a longer interval between the two training sessions (open circles and wide dashed line in **(A)**, only shown for the second training session). Numbers on the y-axis indicate percentage of correctly recognized facial expressions (note that chance level is 25 percent). Error bars indicate SEM. Asterisks indicate significant effects (*p* < 0.05).

Behavioral data are summarized in **Table 1**. Participants showed a significant block-to-block increase of hit rates during both training sessions [*training session 1*, *T*(37) = 1.7, *p* = 0.046, *training session 2*, *T*(37) = 2.9, *p* = 0.033, **Figure 2A]**, and there was no significant difference in learning slopes between training sessions [*training session 1 minus training session 2*, *T*(37) = –0.4, *p* > 0.50 (two-tailed)]. Learning slopes did not differ between the two groups [*2-days interval minus 2-months interval*, *training session 1*, *T*(36) = 0.1, *p* > 0.50 (two-tailed); *training session 2*, *T*(36) = 0.0, *p* > 0.50 (two-tailed)], and there was no interaction between consolidation interval and training session [*T*(36) = 0.1, *p* > 0.50 (two-tailed)]. This indicates that significant learning took place within training sessions, independent of the interval between training sessions.

**Table 1:** Mean hit rates, response times, and unbiased hit rates for all stimuli.

	Hit rate (%)			Response time (ms)	Unbiased hit rate (%)			
	Short videos	Long videos	All videos	All videos	Anger	Disgust	Fear	Sadness
**Training session 1 **
Block 1	48 (±3)	52 (±4)	50 (±2)	982 (±74)				
Block 2	46 (±3)	50 (±3)	50 (±2)	924 (±59)				
Block 3	48 (±3)	58 (±4)	54 (±2)	877 (±59)				
Block 4	44 (±3)	60 (±3)	53 (±3)	859 (±65)				
Block 5	48 (±3)	60 (±3)	53 (±2)	785 (±55)				
**Mean**	**47 (±2)**	**56 (±2)**	**52 (±2)**	**885 (±58)**	**30 (±2)**	**40 (±3)**	**26 (±1)**	**23 (±2)**
**Training session 2**
Block 1	48 (±3)	60 (±2)	55 (±2)	871 (±61)				
Block 2	54 (±3)	60 (±2)	57 (±2)	820 (±56)				
Block 3	54 (±2)	63 (±3)	59 (±2)	825 (±74)				
Block 4	52 (±3)	65 (±2)	59 (±1)	797 (±60)				
Block 5	58 (±3)	67 (±2)	59 (±2)	799 (±66)				
**Mean**	**53 (±3)**	**63 (±2)**	**58 (±1)**	**882**	**35 (±2)**	**48 (±2)**	**31 (+/-1)**	**28 (+/-2)**

*Numbers in brackets indicate SEM (*N* = 38).*

Importantly, there was also a significant increase in hit rates from the first block of the first training session to the first block of the second training session [*T*(37) = 2.6, *p* = 0.007, **Figure 2B**]. Again there was no significant difference between groups [*2-days interval minus 2-months interval*, *T*(36) = –1.2, *p* > 0.10]. This indicates that increased emotion recognition accuracy consolidated across training sessions, independent of the consolidation interval between training sessions.

A similar pattern was observed for response times. There was a significant block-to-block decrease of response times during both training sessions [*training session 1*, *T*(37) = –3.7, *p* < 0.001; *training session 2*, *T*(37) = –2.0; *p* = 0.017], although this decrease was significantly stronger during the first than during the second training session [*training session 1 minus training session 2*, *T*(37) = –2.1, *p* = 0.021]. Learning slopes did not differ between groups in the first training session [*two-days interval minus longer interval*, *T*(36) = 0.3, *p* > 0.50 (two-tailed)], although in the second training session participants with a 2-days consolidation interval showed a stronger decrease of response times than participants in with a 2-months consolidation interval [*2-days interval minus 2-months interval*, *T*(36) = –2.3, *p* = 0.027 (two-tailed)]; this interaction between consolidation interval and training session did not reach statistical significance [*T*(36) = –1.6, *p* > 0.10 (two-tailed)].

Response times decreased significantly from the first block of the first training session to the first block of the second training session [*T*(37) = –2.2, *p* = 0.017] and there was no significant difference between groups [*2-days interval minus 2-months interval*, *T*(36) = 0.4, *p* > 0.30]. Together, these data indicate that response times decreased both within and across training sessions, independent of the consolidation interval between training sessions.

### LONG Vs. SHORT STIMULUS DURATION

**FIGURE 3:**
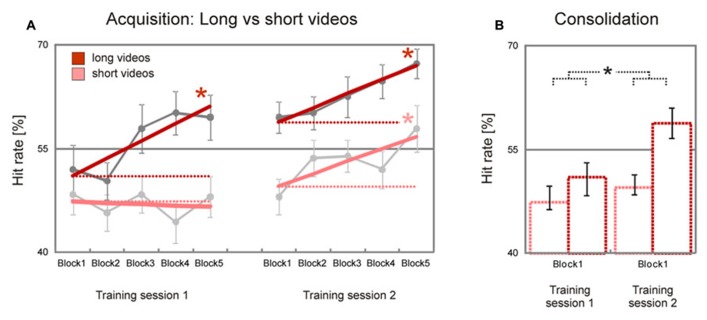
**Improvement of facial emotion recognition accuracy (long vs. short videos).** Participants showed a significant linear increase in emotion recognition accuracy within both training sessions for long videos but not for short videos; for short videos a significant increase in emotion recognition accuracy was observed only in the second training session (dark/bright bold red lines in **A**). In line with this, emotion recognition accuracy increased significantly from the first block of the first training session to the first block of the second training session for long, but not for short videos (dark/bright dashed red lines in **A**, dark/bright bar charts in **B**). Dark/bright gray filled circles in **A** represent block averages for long/short videos across all participants. Numbers on the y-axis indicate percentage of correctly recognized facial expressions (note that chance level is 25%). Error bars indicate SEM. Asterisks indicate significant effects (*p* < 0.05).

As expected, there was a trend for long videos (8–10 s) to be initially recognized less accurately than short videos (2–4 s) [*long minus short videos*, *T*(37) = 1.3, *p* = 0.10]. This difference increased during the first training sessions and remained nearly stable during the second training session: while long videos showed a significant block-to-block increase of hit rates during the first and the second training session [*training session 1*, *T*(37) = 3.0, *p* = 0.002; *training session 2*, *T*(37) = 3.1, *p* = 0.002], short videos showed a significant block-to-block increase of hit rates only in the second training session [*training session 1*, *T*(37) = –0.3, *p* > 0.50; *training session 2*, *T*(37) = 2.2, *p* = 0.017, **Figure 3A**]. The difference between learning slopes for long and short videos in the first, but not in the second, training session was statistically significant [*long minus short videos*, *training session 1*, *T*(37) = 2.1, *p* = 0.021, *training session 2*, *T*(37) = 0.2, *p* > 0.50], with an interaction just below statistical significance [*stimulus duration x training session*, *T*(37) = 1.6, *p* = 0.059]. A similar trend was observed when estimated hit rates during the first blocks of the first and second training sessions were compared [*long minus short videos*, *T*(37) = 1.6, *p* = 0.059, **Figure 3B**]. Together, these data show that initial performance was more accurate for long than for short videos, and that emotion recognition accuracy improved faster for long than for short videos.

### INTERPERSONAL TRAITS

Participants’ IRI scores deviated less than one SD from the norm of their German age reference group (Christoph Paulus, Normentabellen des SPF, Universität des Saarlandes, 2011) on all four subscales (perspective taking, mean = 3.5, SD = 0.6, norm 3.7; fantasy, mean = 3.5, SD = 0.8, norm 3.6; empathic concern, mean = 3.6, SD = 0.7, norm 3.6; personal distress, mean = 2.6, SD = 0.8, norm 2.8).

Overall, correlations between interpersonal traits and initial performance or learning were weak. However, we observed a significant positive correlation between empathic concern and initial hit rates (*y*_1,1_) for long videos [*r* = 0.27, *T*(36) = 1.7, *p* = 0.050 (uncorrected)] and between empathic concern and learning slopes for short videos [*r* = 0.36, *T*(36) = 2.3, *p* = 0.014 (uncorrected)]. Thus, empathic concern predicted both initial performance for long videos and improvement in emotion recognition accuracy for short videos.

### SINGLE EMOTIONS

**FIGURE 4:**
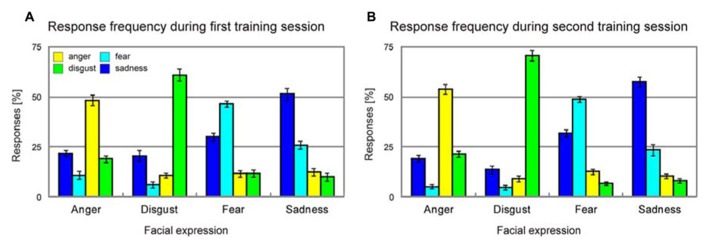
**Response frequency during the first (A) and the second training session (B).** Error bars indicate SEM.

Average unbiased hit rates (Wagner, 1993) showed a significant increase from the first training session to the second training session for each and every emotion [anger, *T*(37) = 2.6, *p* = 0.007; disgust, *T*(37) = 2.9, *p* = 0.003; fear, *T*(37) = 3.2, *p* = 0.001; sadness, *T*(37) = 2.5, *p* = 0.009], and this improvement of emotion recognition accuracy was similar across all emotions [four-by-two ANOVA with factors emotion and training session, emotion × training session interaction, *F*(3,11) = 0.8, *p* > 0.50, **Figure 4**].

## DISCUSSION

We observed a significant improvement of facial emotion decoding skills in healthy adults in a forced-choice emotion recognition paradigm without any external feedback. Participants’ emotion recognition accuracy increased significantly both within and between two training sessions two days to several weeks apart. Although the study population and stimulus sample in the current study were limited to female Caucasian senders and observers, the current study extends previous evidence that facial emotion decoding skills can be significantly and sustainably improved by learning mechanisms that do not rely on external teaching signals.

The neural processes and mechanisms underlying unsupervised learning have extensively been studied in vision research, but improved performance after practice without feedback has also been observed in more cognitive tasks. Below, we discuss similarities and differences between the unsupervised improvement of facial decoding skills observed in the current study, unsupervised perceptual learning of simple stimuli, and other forms of unsupervised learning.

### COMPLEX VERSUS SIMPLE STIMULI

Improvement of perceptual skills after repeated stimulus exposure without external feedback has been most intensively studied in the visual domain (e.g., Karni and Sagi, 1991; Poggio et al., 1992; Crist et al., 1997; more recently Özgen and Davies, 2002), but has also been observed in the auditory (e.g., Goudbeek et al., 2009) and olfactory (e.g., Li et al., 2006) modality. In these studies, participants were typically asked to detect, discriminate or categorize simple visual, auditory or olfactory stimuli. The decision boundary could either be explicitly given (such as “upright” for discrimination of tilted lines) or implicitly defined by the structure of the stimulus set (e.g., for a stimulus set consisting of lines whose tilt angles cluster around 45° and –45° tilt angle, respectively, “upright” can be derived as decision boundary from the structure of the stimulus set). In the first case, stimulus exposure results in enhanced perceptual discrimination along the relevant physical dimension (*perceptual discrimination learning*), particularly around the decision boundary. In the second case, stimulus exposure leads to learning of previously unknown categories (*perceptual category learning*), which in turn can result in perceptual discrimination learning. Both processes could in principle have contributed to the improvement of facial emotion decoding skills observed in the current study. However, the learning problem in the current study differed from that in studies using simple visual or auditory stimuli in at least two important factors: First, the physical feature space spanned by the facial emotional expressions used in the current study comprised far more dimensions than the space spanned by the simple stimuli used in previous studies. Second, participants in the current study had extensive prior (perceptual and semantic) knowledge about the categorical structure underlying the stimulus space.

### PHYSICAL FEATURE SPACE AND PRIOR KNOWLEDGE

Recent studies show that humans easily learn new stimulus categories without feedback if these categories are defined by a single physical dimension (such as tilt angle), but are surprisingly inept in learning perceptual categories without an external teaching signal if learning requires the integration of two or more perceptual dimensions (such as tilt angle *and *length (*information integration learning*); Ashby et al., 1999; Goudbeek et al., 2009). This suggests that prior category knowledge might play an important role in facial emotion recognition learning.

In further support of this, a study on chimpanzee facial emotion recognition found that human observers perceived prototypical chimpanzee (*Pan troglodytes*) facial expressions categorically if they had previously learned (nonsense) verbal labels for each category (Fugate et al., 2010), while extensive perceptual experience with non-human primate facial expressions alone did not result in categorical perception (it should be noted though that participants in that study were also counted as having perceptual expertise if they had prior experience with a primate species other than chimpanzees). Another study on visual category learning found that semantic category knowledge can help to direct attention to relevant stimulus dimensions (Kim and Rehder, 2011).

In addition to semantic category knowledge, innate or learned perceptual knowledge might play an important role in facial emotion recognition learning. Specifically, innate or acquired neural algorithms that favor processing along biologically relevant higher-order perceptual dimensions (e.g., anger–disgust, anger–fear, anger–sadness, disgust–fear, disgust–sadness, fear–sadness) rather than physical dimensions (e.g., form and relative spacing of lips, brows, and eyes) could substantially reduce the dimensionality of the relevant perceptual space and thereby facilitate unsupervised learning. Empirical support for the assumption that such algorithms indeed develop early in life comes from the observation that infants, but not adults, readily learn multidimensional speech–sound categories by mere exposure (Maye et al., 2002; Goudbeek et al., 2009). In the current study, learning was facilitated both by empathic abilities and initial performance.

One important task for future studies will be to examine the effects of prior (learned or innate) semantic or perceptual knowledge on unsupervised learning of facial emotion decoding skills. This is particular interesting as observers will likely have less prior knowledge about the emotional behavior of senders who have a different social, cultural or ethnic background than the observer.

### SPECIFIC VERSUS GENERALIZED LEARNING

Early studies on perceptual learning using simple physical stimuli in the visual domain found that training effects were remarkably specific to the particular stimuli used for training (e.g., an increased ability to discriminate distances between vertical lines did not generalize across line orientation or even visual location, Poggio et al., 1992; Crist et al., 1997). This has been taken as evidence that perceptual learning can take place very early in the visual processing stream (Gilbert, 2001). Thus the question arises whether the improvement of facial decoding skills observed in the current study is limited to the particular sample of individuals seen during training or whether it generalizes beyond individual senders and maybe even sensory modalities.

Interestingly, there is accumulating evidence from neuroimaging studies that improved perceptual performance can be related to neural changes at different cortical levels, possibly depending on the particular perceptual task (Schoups et al., 2001; Schwartz et al., 2002; Furmanski, 2004; Little and Thulborn, 2005; Sigman et al., 2005; Li et al., 2006; Op de Beeck et al., 2006; Jiang et al., 2007; Law and Gold, 2008; van der Linden et al., 2008; Yotsumoto et al., 2008; Li et al., 2009; Wong et al., 2009; Zhang et al., 2010; Kahnt et al., 2011; Folstein et al., 2012; Myers and Swan, 2012), and that neural changes in higher cortical areas are associated with less specific learning effects (for review, see Sasaki et al., 2010). Extrapolating this evidence to the current study one might propose that if improved facial emotion decoding skills are related to neural plasticity in higher visual areas [e.g., occipito-temporal areas that support facial emotion recognition independent of facial identity (Fox et al., 2009)], then these learning effects should generalize beyond individual senders. Even more interestingly, one might ask whether learning effects can also generalize across sensory modalities. For example, it would be highly interesting to see whether perceivers who become more accurate at discriminating between facial emotional expression of different categories would at the same time become more accurate at discriminating vocal emotional expressions of the same categories (see Shams et al., 2011 for a related account). This would point towards increased discrimination accuracy at a neural level that receives input from different sensory modalities. Further combined behavioral and neuroimaging studies are needed to address these questions.

### ACTIVE DECISION MAKING AND STIMULUS SALIENCE

Another factor that might have an important effect on unsupervised learning of facial decoding skills is explicit decision-making versus passive observation. One of the first reports of perceptual learning is the observation that passive exposure to visual stimuli can increase visual discrimination in rats (Gibson and Walk, 1956). In most perceptual learning studies in humans, participants were required to actively make a decision, but there are also a few studies that report perceptual learning after mere stimulus exposure in humans (e.g., Skrandies and Fahle, 1994). Although these findings suggest that explicit decision making is not essential for perceptual learning to occur, active decision making could still act as an enhancing factor. In a recent review, Sasaki et al. (2010) underline the role of signal strength in perceptual learning, and there is evidence that if participants are required to make a decision in the absence of external feedback an internal error signal is created that can serve as reinforcement signal and thereby facilitate learning (Daniel and Pollmann, 2012). Similarly, emotional salience might act as an internal signal amplifier and thereby facilitate learning in real life. Empirical evidence for this comes from a series of studies of physically abused children that showed that abused children recognize angry facial expressions more rapidly than controls (Pollak et al., 2009). Furthermore, compared to healthy controls, abused children’s category boundaries for angry expressions were shifted towards fearful and sad facial expressions (Pollak and Kistler, 2002). Although these studies do not allow to completely separate effects of emotional salience from effects of frequent exposure they provide some evidence that emotional salience might play a role in learning of facial emotion recognition. Behavioral studies that closely model real life situations are needed to investigate the role explicit decision making, salience, and related factors in more detail.

### OTHER FORMS OF UNSUPERVISED LEARNING

In a study on auditory perceptual learning, Hawkey et al. (2004) distinguished between *perceptual learning* (which refers to performance changes, “brought about through practice or experience, that improve an organism’s ability to respond to its environment”, p. 1055) and *procedural learning* (which refers to “improvement in performance on a task that results from learning the responds demands of the task”, p. 1055). In the current study, *procedural learning* would refer to any improvement in performance that is not specific for facial emotional expressions (or, in fact, for any expressive emotional behavior, see below) but for features of the particular experimental set-up used in the current study, e.g., selecting and pressing the appropriate response button on a keyboard. Another possible factor that might confound results in studies that require participants to repeatedly classify stimuli into a number of predefined categories is that over the course of the experiment participants might acquire knowledge about a particular stimulus set (e.g., the frequency distribution of stimuli of a particular class) which could help them to develop response strategies that increase performance in the absence true stimulus-related learning (see e.g., Scherer and Scherer, 2011).

In the current study, we partly controlled for procedural learning by switching response buttons across training sessions. A more stringent control that should certainly be implemented in future studies would be to test the participants’ facial decoding skills after training on a completely different experimental set-up (e.g., by showing the participants static images rather than videos and asking them to respond orally rather than via a computer).

Improved performance after practice without feedback has also frequently been observed in more cognitive tasks, for example when participants are tested on cognitive abilities (e.g., Hausknecht et al., 2002, 2007). A number of factors have been discussed to explain increased performance in such tasks, the most relevant for the current observation perhaps being reduced anxiety and increased motivation. Although these factors are probably more important in settings where participants know or have the impression that they being assessed for their personal abilities, future studies on facial decoding skills should include additional affective and motivational state questionnaires to control for these factors.

## CONCLUSION

In sum, the current study extends previous evidence that facial emotion decoding skills can be significantly and sustainably improved by learning mechanisms that do not rely on an external teaching signal. Such mechanisms might provide a basis for dynamic, life-long tuning of facial emotion decoding skills in humans. Importantly, the particular pattern of improvement of facial decoding skills observed in the current study, i.e., dependency of learning on stimulus duration and empathy-related personally traits, are difficult to explain by any confounding factors. Nevertheless, the results of the current study call for further systematic behavioral and neuroimaging studies that investigate (i) the degree to which unsupervised learning of facial emotion decoding skills relies on prior semantic and perceptual knowledge (ii) the degree to which improved emotion recognition generalizes across senders and sensory modalities, (iii) possible modulating effects of explicit decision making and stimulus salience and (iv) control more stringently for confounding effects. Such studies will, hopefully, (i) allow to develop efficient training programs to improve cross-cultural emotion decoding skills and (ii) draw the attention of the neuroscience community to the role of neural plasticity in human social behavior.

## AUTHOR CONTRIBUTIONS

Silke Anders, Jan O. Huelle, and Benjamin Sack conceived the experiment; Benjamin Sack and Katja Broer acquired data; Silke Anders, Irina Komlewa, and Benjamin Sack analyzed the data; Silke Anders wrote the manuscript; all authors edited the manuscript.

## Conflict of Interest Statement

The authors declare that the research was conducted in the absence of any commercial or financial relationships that could be construed as a potential conflict of interest.
